# Effect of lactic acid fermentation on the nutritional quality and consumer acceptability of African nightshade

**DOI:** 10.1002/fsn3.2912

**Published:** 2022-04-26

**Authors:** Frank Sangija, Haikael Martin, Athanasia Matemu

**Affiliations:** ^1^ Department of Food Biotechnology and Nutritional Sciences Nelson Mandela African Institution of Science and Technology Arusha Tanzania

**Keywords:** African nightshade, antinutrients, consumer acceptability, fermented pickle, minerals, relish, vitamin C, β‐carotene

## Abstract

African nightshade (ANS) is among many underexploited and neglected indigenous vegetables. This study assessed the effect of lactic acid fermentation (LAF) on nutritional and sensory quality in *Solanum villosum* (Sv) and *Solanum scabrum* (Ss). Spontaneously fermented (SF) and controlled fermented (CF) conditions using *Lactobacillus plantarum* LP90 and *Leuconostoc mesenteroides* LM58 were employed for 15 days and 120 h. From the fermented pickles, relish products were prepared using cooking oil and a variety of spices. The relish products were subjected to a consumer acceptability test. Results show a significant drop in pH to <3.5, increasing titratable acidity (TTA) to around 0.6 after 120 h and 15 days of CF and SF, respectively. LAF resulted in a 2.6–5 and 1.6–4.8‐fold significant rise in β‐carotene in pickles and their relish products. All pickles and relish products exhibited a significant decrease (*p <* .05) in vitamin C by 88.33%–95.90%. LAF significantly reduced total phenolic (26%– 43%) and Chlorophyll (16.45%–39.25%). On the other hand, LAF showed improvement in minerals content (P, Ca, Fe, and Zn) and reduction of tannin (76.27%–92.88%) and oxalate (77.33%–90%) levels. LAF relish products were highly preferred by the consumers, with *S. villosum* controlled fermented relish (SvCFR) leading. All fermented relishes were stable at ambient (27°C) and refrigeration (4°C) temperatures after 6 months of storage. Generally, LAF is an effective method for ANS preservation, with improved nutritional quality and safety. LAF can therefore be recommended to small‐scale farmers, processors, and households for ANS preservation. Ultimately, this method enhances the nutrition and sensory quality, safety, and livelihood.

## Graphical abstract

Lactic acid fermentation (LAF) improved β‐carotene and minerals contents but reduced vitamin C, total phenols, and Chlorophyll levels. Also, it reduces antinutrients, tannin, and oxalates. Relish products were stable at ambient and refrigeration temperatures after six months of storage and consumers preferred all relish products.

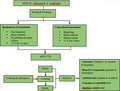

## 1 INTRODUCTION

African nightshades (ANSs) are among widely distributed and consumed African indigenous vegetables (AIVs) in Tanzania (Keller, 2004; Weinberger & Msuya, 2004). *Solanum scabrum*, *Solanum villosum*, *Solanum americanum*, and *Solanum nigrum* are commonly available species in Tanzania. African nightshades (ANSs) are a good source of essential minerals (K, P, Ca, Fe, and Zn), vitamins C, and β‐carotene. Also, they contain bioactive compounds such as lutein, zeaxanthin, polyphenol, flavonoids and have medicinal properties (Odongo et al., 2018; Sangija et al., 2021). Studies show the contribution of ANS in improving the income and food security in African households (Abukutsa‐Onyango, 2010; Sangija et al., 2021). Of recent development, there has been a decline in ANS utilization caused by a shift toward exotic vegetables, which consumers rank as nutritious and high‐yielding. Postharvest losses and poor postharvest handling have also significantly constrained ANS utilization in sub‐Saharan Africa (SSA) (Sangija et al., Unpublished). In Tanzania, like other SSA countries, the postharvest loss of ANS is a significant problem confronting many AIVs. Also, losses contribute to high prices and shortages of ANS in the offseason and wastage during peak season (Abukutsa‐onyango, 2015; Habwe & Walingo, 2008; Sangija et al., 2021). Furthermore, pests and diseases negatively impact ANS utilization (Ondieki et al., 2011; Sangija et al., 2021).

There are various local preservation methods of AIVs, including blanching, stir‐frying, sun‐drying, solar drying, and fermentation (Sangija et al., 2021; Wafula, 2017). Fermentation is one of the oldest technologies used to extend shelf life, improve palatability, and enhance nutrition and sensory quality of food (Ray & Didier, 2014). During fermentation, lactic acid bacteria (LAB) utilize sugar and other nutrients to produce lactic acid (LA), acetic acids, and hydrogen peroxide, preserving the foods and extending their shelf life (Sangija et al., 2021). Although fermentation reduces water‐soluble vitamins (B1 and B2), it still leaves a significant amount of vitamin C and β‐carotene to supplement the recommended daily intake (Wafula, 2017). Fermentation can either occur naturally, i.e., spontaneously or under controlled conditions. The use of starter cultures, such as *Leuconostoc mesenteroides*, *Lactobacillus fermentum*, *Lactobacillus plantarum*, *Lactobacillus acidophilus*, and *Lactococcus lactis* in ANS fermentation, has been reported (Owade et al., 2021; Wafula, 2017). This study aimed to determine the effect of LAF on nutritional quality, safety, and sensory quality of ANS to minimize postharvest losses.

## 2 MATERIALS AND METHODS

### 2.1 Materials

Africa nightshades (ANSs) (*S*. *villosum* and *S*. *scabrum*) were collected in Arumeru District, Arusha, Tanzania. The leaves were transported under cold storage to the Food Processing Unit at the Nelson Mandela African Institution of Science and Technology (NM‐AIST) for further processing. Other ingredients, such as cooking oil, onions, and a variety of spices (garlic, pepper, cardamom, turmeric, cumin, clove, and cinnamon), were purchased in Arusha, Tanzania. Freeze‐dried probiotic *Lactobacillus plantarum* LP90 (100 billion CFU (colony‐forming units)/g) and *Leuconostoc mesenteroides* LM58 (100 billion CFU/g) were obtained from the Wuhan Healthdream Biological Technology Co., Ltd. The starter culture was stored in the freezer at −24°C.

### 2.2 African nightshade leaves preparation

The freshly harvested *S*. *scabrum* (Ss) and *S. villosum* (Sv) leaves were sorted, washed, and then placed in a perforated vessel for water drainage before weighing. For controlled fermentation (CF), the leaves were blanched at 90°C for 5 min and immediately cooled in ice water (De Corcuera et al., 2004).

### 2.3 Fermentation of African nightshade leaves and relish preparation

In CF, blanched Ss and Sv leaves were submerged into a brine solution (4% salt and 2% sugar), and *L*. *plantarum* LP90: *L*. *mesenteroides* LM58 (1:1) were inoculated separately according to Wafula (2017) with slight modification. The mixture was incubated at 25°C for 120 h. After 120 h of controlled fermentation, Ss (*S. scabrum* controlled fermented (SsCFP)) and Sv (*S. villosum* controlled fermented (SvCFP)) pickles were obtained. In SF, unblanched Ss and Sv leaves were likewise submerged into a brine solution (4% salt and 2% sugar) and incubated at 25°C for 15 days (Wafula, 2017) with some modifications. After 15 days, spontaneously fermented pickles were obtained (*S. scabrum* spontaneous fermented pickle (SsSFP) and *S. villosum* spontaneous fermented pickle (SvSFP). The pH and titratable acidity (TTA) were recorded daily for SF and CF.

Pickle products SsSFP and SvSFP from SF and (SsCFP and SvCFP) from CF were cooked for 10 min mixing with cooking oil, onions, and different spices (garlic, pepper, cardamom, turmeric, cumin, clove, and cinnamon) employed in relish‐making. Four relish products of *S*. *scabrum* controlled fermented relish pickle (SsCFRP), *S. villosum* controlled fermented relish pickle (SvCFRP), *S. scabrum* spontaneous fermented relish pickle (SsSFRP), and *S. villosum* spontaneous fermented relish pickle (SvSFRP), respectively. The relishes were then cooled, packaged in PET bottles, and stored at refrigeration and ambient temperatures for shelflife study.

### 2.4 Determination of titratable acidity

Titratable acidity (TTA) was determined, as described by Rajković et al. (2007). About 10 ml of brine solution from spontaneously and controlled fermented Ss and Sv was mixed with 10 ml of distilled water in a conical flask. The mixture was titrated with 0.1N NaOH using two drops of phenolphthalein indicator (LOBA Chemie, India) to a persistent pink end. TTA was calculated in terms of LA anhydrous as follows:

TTA(%)=0.09×Titer(ml)

### 2.5 Determination of vitamin C

The total vitamin C content of the fresh leaves, pickle, and relish products was determined (Kapur et al., 2012). About 10 g of sample was mixed with 50 ml of 3% metaphosphoric acid and 8% acetic acid solution (LOBA Chemie, India) and centrifuged at 4000 rpm (revolutions per minute) (centrifuge 5810, Germany) at ambient temperature for 15 min. Four milliliters (4 ml) of the extract was treated with 0.23 ml of bromine water (3%) (LOBA Chemie, India), followed by the addition of 0.13 ml of 10% thiourea solution (LOBA Chemie, India), and then 1 ml of 2,4 dinitrophenylhydrazine solution (LOBA Chemie, India). The mixture was incubated in a thermostatic water bath at 37°C for 3 h. The mixture was then cooled for 30 min and then treated with 6 ml of chilled 85% sulfuric acid (LOBA Chemie, India). The absorbance of the resulting red‐colored solution was measured using a Spectrophotometer (Heidolph, Germany) at 521 nm. The total ascorbic acid content was estimated, based on the standard curve of ascorbic acid, and the result was expressed as mg/100 g (wet basis).

### 2.6 Determination of β‐carotene

The β‐carotene was determined as described by Perez‐Lopez (2010) and Association of Official Agricultural Chemists (AOAC) (1980) with some modifications. Five grams (5 g) of the sample (raw, pickle, and relish products) in 1% butylated hydroxytoluene (BHT) (LOBA Chemie, India) was homogenized using a blender (Moulinex, China) for 5 min and placed into the falcon tube (BR Biochem, India). The homogenate was treated with 30 ml of solvent (50% hexane:25% acetone:25% ethanol) (LOBA Chemie, India) and then centrifuged at 4000 rpm at ambient temperature for 10 min. The supernatant was collected and filtered using Whatman No. 1 filter paper (Whatman International Ltd., India), and then was re‐extracted two times until the residue was colorless. The supernatant was combined, and the volume was recorded. The supernatant was saponified with 40% potassium hydroxide (LOBA Chemie, India) and left at ambient temperature for 12 h. The mixture was then transferred into the separating funnel and treated with the same volume of 10% w/v sodium chloride (LOBA Chemie, India) for removing moisture. The mixture was then shaken vigorously, and the upper phase was collected and dried over anhydrous sodium sulfate (LOBA Chemie, India). The β‐carotene content was estimated based on the standard curve of β‐carotene, and the result was expressed as mg/100 g (wet basis).

### 2.7 Determination of Chlorophyll

Chlorophyll content of the raw leaves and pickle products was determined as described by Su et al. (2010), with some modifications. About 1.0 g of sample was taken and ground into a fine pulp using mortar and pestle with about 10 ml of 80% acetone (LOBA Chemie, India). The pulp was centrifuged at 4000 rpm at ambient temperature for 5 min, and the green supernatant was then transferred to a 50 ml volumetric flask for Chlorophyll determination. The sediment in the centrifuge tube was scrapped and ground with the same mortar and pestle with a small amount of 80% acetone (LOBA Chemie, India) to extract the residual Chlorophyll. The mixture was centrifuged at 4000 rpm at ambient temperature for 5 min; the supernatant was then mixed with the previous supernatant into the volumetric flask. Re‐extraction was done until no perceptible green color was left in the residue. The supernatant was made to a 50 ml volumetric flask with 80% acetone (LOBA Chemie, India). The extract was placed into the refrigerator for 10 min to lower the temperature. The absorbance of the extract was read at 663 and 645 nm using a Spectrophotometer (Heidolph, Germany), and 80% acetone was used as the blank. The amount of Chlorophyll was calculated using the empirical formula:

$$\mathrm{Chlorophyll}\;a,\;\mathrm{mg}/g\;\mathrm{tissue}\;=12.7\;\left(A663\right)‐2.69\left(A645\right)\times \frac{V}{100X\;W}$$

$$\mathrm{Chlorophyll}\;b,\;\mathrm{mg}/g\;\mathrm{tissue}=22.9\;\left(A645\right)‐4.68\left(A663\right)\times \frac{V}{100X\;W}$$

ChlorophyllTotal,mg/gtissue=Chlorophylla+Chlorophyllb=20.31OD645+8.05OD645

Where A = absorbance at specific wavelengths; V = final volume of chlorophyll extract; W = fresh weight of the tissue extracted.

### 2.8 Determination of the total phenolic content

The total phenolic content of raw Ss and Sv, pickle, and relish products was determined by the Folin–Ciocalteu method (Mahdavi et el., 2011). Homogenized samples (10 g) were mixed with 30 ml of 80% methanol (LOBA Chemie, India) and centrifuged at 3000 rpm at ambient temperature for 15 min. The residue was re‐extracted twice. Precisely, 1 ml of the methanol extract was diluted 10 times with the extraction solvent. An aliquot (0.5 ml) of the diluted sample was mixed with 2.4 ml of deionized water, 2 ml of sodium carbonate (2%) (LOBA Chemie, India), and 0.1 ml of Folin–Ciocalteu reagent (FCR) (LOBA Chemie, India). The mixture was incubated in a dark place at ambient temperature for 60 min, and the absorbance was determined at 750 nm using a Spectrophotometer (Heidolph, Germany).

### 2.9 Determination of the mineral content

Minerals (P, S, Ca, Fe, Zn, Cu, Mn) were analyzed according to the method of (Croffie et al., 2020). The samples (raw leaves, pickle, and relish products) were dried at 60°C in the conventional oven (UN30/Germany). The samples were ground using mortar and pestle and sieved in a sieve of 60‐micron size (Shanghai sieves/ China). Four grams of the sample was mixed with 0.9 g of binder (Hoechstwax) (Cereox fluxana‐BM‐0002‐1/Germany) using a pulverizer (PulverisetteTM/Germany) at the speed of 150 revolutions per sec for 30 min. The mixture was then compressed to form a pellet using a hydraulic pressing machine (Vaneox Fluxana PP25, Germany) at 15 psi (pounds per square inch). The Energy‐Dispersive X‐ray Fluorescence (EDXRF) (Xlab pro‐Spectro xepos Spectrometer/Germany) was used to quantify the mineral content. The minerals reading was corrected using spinach leaves standard/Standard Reference Materials (SRM) 1570a, from the National Institute of Standards and Technology (NIST).

### 2.10 Antinutrients’ determination

#### 2.10.1 Oxalates’ determination

The oxalate content in raw leaves, pickles, and relish products was determined by the titration method, as described by Agbaire (2011). In one (1 g) sample, 75 ml of 3 M sulfuric acid was added while stirring using a magnetic stirrer for 60 min. The mixture was filtered using Whatman filter paper No. 1 (Whatman International Ltd, India). About 25 ml of the filtrate was titrated while hot against 0.05 M potassium permanganate solution (LOBA Chemie, India), until a faint pink color persisted for at least 15–30 s. The oxalate content was calculated by taking 1 ml of 0.05 M potassium permanganate as equivalent to 2.2 mg oxalate.

$$O=\frac{Ts\;\times \;Md\;\times \;Mo\;\times \;100}{\mathrm{Ws}}$$

Where, O = oxalate concentration in mg/100 g, Ts = volume of potassium permanganate used for the sample, Md = number of moles of potassium permanganate reacted, Mo = number of moles of oxalate reacted, and Ws = sample weight.

##### Determination of tannin

The tannins were determined by the Folin–Ciocalteu method according to Chandran and Indra (2016). A 10 g of homogenized sample (raw ANS, pickle, and relish) was mixed with 30 ml of 80% methanol (LOBA Chemie, India) and centrifuged at 3000 rpm at ambient temperature for 15 min. The residue was re‐extracted twice. Precisely, 1 ml of the methanol extract was diluted 10 times with the extraction solvent. An aliquot (0.5 ml) of the diluted sample was mixed with 7.5 ml of distilled water, 1 ml sodium carbonate (35%) (LOBA Chemie, India), and 0.5 ml of Folin–Ciocalteu reagent (FCR) and diluted to 10 ml with distilled water (LOBA Chemie, India). The mixture was shaken well and kept at room temperature for 30 min. The tannic acid standard was used to prepare the standard curve. Absorbance for test and standard solutions was measured against the blank at 700 nm with a Spectrophotometer (Heidolph, Germany). The tannin content was expressed in mg of tannic acid equivalents/100 g of wet basis.

### 2.11 Microbial determination of relish products

Total bacteria, fungal counts, coliform, and *Lactobacillus* were analyzed separately by the pour plate technique as described by Karami et al. (2017), Rahimi et al. (2019), and Singh et al. (2013) with slight modification. Briefly, 1 g of homogenized relish products was mixed with 9 ml of sterile peptone salt (LOBA Chemie, India) and vortexed for 5 min. Then, 1 ml of the suspension was transferred into the peptone salt diluents (9 ml) up to 10^–4^ dilution series. From each dilution, 1 ml of diluted sample was inoculated into the respective growth media; total bacteria (plate count agar), yeast and mold (potato dextrose agar), *Lactobacillus* (de Man, Rogosa, and Sharpe (MRS) agar), and coliform (violet red bile agar) (HiMedia Laboratories, Mumbai, India). The incubation conditions were: bacteria (30°C, 24 hr), molds and yeasts (37°C, 72 hr), coliforms (30°C, 48 hr), and *Lactobacillus* (37°C, 48 hr) at anaerobic conditions.

### 2.12 Shelflife test of the relish products

The shelflife study of relishes was conducted at refrigeration (4°C) and ambient temperatures according to Pala and Agnihotria (1994), with some modifications. Relishes in transparent polyethylene (PET) plastic‐capped bottles were kept at refrigeration (4°C) and ambient temperatures for 6 months consecutively. Monthly monitoring of TTA, pH, total bacteria, LAB, yeast, and mold was performed.

### 2.13 Consumer acceptability test

The consumer acceptability test was conducted in the Moshi District Council (Moshi rural) in Kilimanjaro and Morogoro District Council (Morogoro rural) in Morogoro regions with high and low ANS production. About 370 untrained panelists, mainly ANS consumers, participated in the sensory evaluation of the relish products. They were selected based on their history of consumption of ANS. Four relish products, i.e., *S. scabrum* spontaneous fermented relish (SsSFR), *S. scabrum* controlled fermented relish (SsCFR), *S. villosum* spontaneous fermented relish (SvSFR), and *S. villosum* controlled fermented relish (SvCFR), were tasted for consumer acceptability. A 9‐scale Hedonic test was used (Yang & Lee, 2018), with 1 = dislike extremely, 5 = neither like nor dislike, and 9 = like extremely. Panelists were served with all four coded relish products and filled out an evaluation form after tasting.

### 2.14 Statistical analysis

Experiments were conducted in triplicate, and data were expressed as mean values ± *SD*. SPSS software version 25 was used for data processing. The one‐way analysis of variance (ANOVA) and post hoc test (least significant difference) were used to compare the mean between the species of *Solanum* for the fermented pickles and relish products, and the differences were considered significant when *p* < .05.

## 3 RESULTS AND DISCUSSION

### 3.1 Effect of LAB on African nightshades

African nightshades (ANSs) (*S*. *scabrum* and *S. villosum*) at an initial pH of 7.4 were fermented spontaneously and under controlled conditions for 15 days and 120 h, respectively. A sharp drop in pH to 4.7 and pH to 3.5 was observed within 24 h for both spontaneous fermentation (SF) and controlled fermentation (CF), respectively (Table 1). Furthermore, a slight pH drop in SvCFP and SsCFP has observed a similar trend. A sharp pH drop indicates the ability of acidification by LAB during fermentation (Stoll et al., 2021). LAB produces various organic acids, with predominant lactic acid (LA), hence the preservation effect (Degrain et al., 2020). A combination of *L. plantarum* LP90 and *L. mesenteroides* LM58 in 3% brine solution resulted in a sharp pH drop. Therefore, faster, more profound, stable, and more controlled fermentation can be achieved using starter cultures (Stoll et al., 2021). Similar studies on the use of commercial starter cultures, such as *Lactiplantibacillus plantarum* BFE 5092, *Limosilactobacillus fermentum BFE* 6620, *Lactobacillus plantarum* (17a), *Weissella cibaria* (21), *Leuconostoc pseudomesenteroides* (56), *W. cibaria* (64), or *L. plantarum* (75), in ANS fermentation had been reported (Degrain et al., 2020; Stoll et al., 2021; Wafula, 2017). Heterofermenters, mainly *Leuconostoc mesenteroides* and *L. brevis,* can initiate the fermentation process, while *L. plantarum* occur later (Belitz et al., 2009; Hutkins, 2006; Sangija, 2021).

**TABLE 1:** Changes in titratable acidity (TTA) and pH during spontaneous and controlled fermentation of African nightshade (ANS)

Days	Parameter	SvSFP (SF)	SvCFP (CF)	SsSFP (SF)	SsCFP (CF)
0	pH	7.4 ± 0.1^a1^	7.4 ± 0.1^a1^	7.4 ± 0.1^a1^	7.4 ± 0.1^a1^
	TTA	0.072 ± 0.00^aA^	0.072 ± 0.00^aA^	0.045 ± 0.00^bA^	0.045 ± 0.00^bA^
1	pH	4.5 ± 0.1^a2^	3.5 ± 0.2^b2^	4.7 ± 0.3^a1^	3.5 ± 0.2^b2^
	TTA	0.2 ± 0.01^aB^	0.37 ± 0.01^bB^	0.1 ± 0.00^cB^	0.4 ± 0.01^bB^
2	pH	3.7 ± 0.1^a3^	3.4 ± 0.2^a2^	4 ± 0.2^b3^	3.4 ± 0.1^a2^
	TTA	0.24 ± 0.02^aB^	0.42 ± 0.03^bC^	0.18 ± 0.02^cC^	0.52 ± 0.03^dC^
3	pH	3.5 ± 0.2^a3^	3.4 ± 0.1^a2^	4 ± 0.0^b3^	3.4 ± 0.0^a2^
	TTA	0.3 ± 0.01^aC^	0.56 ± 0.03^bD^	0.2 ± 0.01^cC^	0.54 ± 0.02^bC^
4	pH	3.4 ± 0.1^a3^	3.3 ± 0.2^a2^	3.9 ± 0.1^b3^	3.3 ± 0.2^a2^
	TTA	0.34 ± 0.00^aC^	0.61 ± 0.01^bE^	0.25 ± 0.02^cCD^	0.56 ± 0.02^dC^
5	pH	3.4 ± 0.1^a3^	3.2 ± 0.1^a2^	3.8 ± 0.0^b3^	3.2 ± 0.0^a2^
	TTA	0.37 ± 0.00^aD^	0.64 ± 0.00^bE^	0.27 ± 0.01^cD^	0.62 ± 0.02^bD^
6	pH	3.4 ± 0.0^a3^		3.7 ± 0.2^b3^	
	TTA	0.42 ± 0.02^aE^		0.3 ± 0.04^bDE^	
7	pH	3.4 ± 0.0^a3^		3.6 ± 0.2^a34^	
	TTA	0.51 ± 0.01^aF^		0.33 ± 0.01^bE^	
8	pH	3.3 ± 0.0^a3^		3.5 ± 0.3^a34^	
	TTA	0.52 ± 0.02^aF^		0.35 ± 0.02^bE^	
9	pH	3.3 ± 0.2^a3^		3.5 ± 0.0^a4^	
	TTA	0.57 ± 0.03^aG^		0.37 ± 0.01^bEF^	
10	pH	3.3 ± 0.0^a3^		3.4 ± 0.2^a34^	
	TTA	0.6 ± 0.02^aG^		0.4 ± 0.00^bF^	
15	pH	3.3 ± 0.1^a3^		3.3 ± 0.1^a34^	
	TTA	0.64 ± 0.02^aG^		0.68 ± 0.04^aG^	

Note

Mean value (*n* = 3) ± *SD*. Means with different superscript small letters within a row (pH and TTA) are significantly different *p* < .05. Means with different superscript capital letters within a column (TTA) are significantly different *p* < .05. Means with different superscript numbers within a column (pH) are significantly different *p* < .05.

Abbreviations: SvSFP, *S. villosum* spontaneous fermented pickle; SvCFP, *S. villosum* controlled fermented pickle; SsSFR, *S*. *scabrum* spontaneous fermented relish; SsCFR, *S. scabrum* controlled fermented relish.

On the other hand, a steady pH drop was observed in SF, resulting in a prolonged fermentation time with a pH of 3.5 attained in 72 h for SvSFP and 192 h for SsSFP, respectively (Table 1). Naturally occurring bacteria grow over 1–2 weeks to produce lactic acid (LA), whereas added salt controls the type and rate of the fermentation (Behera et al., 2020). In this study, the addition of brine solution (4% salt and 2% sugar) facilitated the attainment of an optimal pH of 3.5 within 3–8 days of spontaneous fermentation (Table 1).

Similarly, a sharp increase in TTA to 0.4 within 24 h was observed in CF, instead of SF (Table 1). Wafula (2017) reported an increase in TTA due to ANS fermentation. Lactic acid (LA) production prevents the growth of food poisoning bacteria and other spoilage microorganisms, thus enhancing product safety (Behera et al., 2020) and sensory quality.

### 3.2 Effect of fermentation on the nutritional quality

**FIGURE 1:**
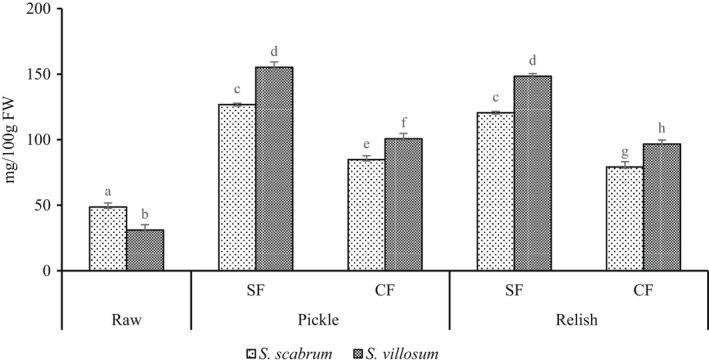
β‐Carotene content of fermented African nightshade (ANS) pickle and relish products. Pickle (*S. scabrum* spontaneous fermented pickle (SsSFP), *S. villosum* spontaneous fermented pickle (SvSFP), *S. scabrum* controlled fermented pickle (SsCFP), and *S. villosum* controlled fermented pickle (SvCFP)) and relish (*S*. *scabrum* spontaneous fermented relish (SsSFR), *S. villosum* spontaneous fermented pickle (SvSFP), *S. scabrum* controlled fermented relish (SsCFR), and *S*. *villosum* controlled fermented relish (SvCFR)). Whereas; Ss, *S*. *scabrum*; Sv, *S. villosum*; CF, controlled fermentation; SF, spontaneous fermentation. Means ± *SD* (*n* = 3) with different superscript letters represent significant difference at *p* < .05

The results on β‐carotene content in LAF are presented in Figure 1. LAF significantly increased the β‐carotene content in pickle and relish products. The β‐carotene content in fresh *S. scabrum* and *S. villosum* was 48.7 mg/100 g and 31.1 mg/100 g, respectively (Figure 1). A 2.6–5‐fold significant increase in β‐carotene in pickles and 1.6–4.8‐fold in relishes were reported (Figure 1). For pickles, *S. villosum* exhibited significantly higher (*p* < .05) β‐carotene values than *S. scabrum* as opposed to their relish products. Likewise, for relish products, *S. villosum* had significantly high β‐carotene, unlike *S. scabrum* (Figure 1). Fermentation retained a substantial amount of β‐carotene (Wafula, 2017). Also, *Lactobacillus gasseri* fermentation of carrot juice increased β‐carotene (Xu et al., 2020). Chinese cabbage showed increased β‐carotene after four days of spontaneous fermentation (Chavasit et al., 2002), similar to the study findings. The high increase was due to the extraction of carotene from the stable lipoprotein complexes during fermentation. Similarly, Bartkiene et al. (2015) reported a 43.9% average β‐carotene increase in LA fermented tomato powder compared with the nonfermented samples. The carbon source is one of the significant factors that influence the production of carotenoids in the complex enzymatic system used by microorganisms (bacteria, yeasts, and molds). Carotenogenesis can be affected by temperature, pH, reactive oxygen species (ROS), and the oxidative reaction of ultraviolet (UV) light and transition metals (Mapelli‐Brahm et al., 2020). Fermentation disrupts the plant matrix and cell cluster, resulting in carotenoid liberation and bioaccessibility but can undergo oxidation or degradation. Furthermore, some LAB strains possess enzyme activity that favors carotenoid extraction (Mapelli‐Brahm et al., 2020). Also, oxidative stress has been reported to induce the production of β‐carotene, γ‐carotene, and lycopene in *B*. *trispora*. Therefore, LAF increased β‐carotene content from 30% to 40% in cabbage (Mapelli‐Brahm et al., 2020).

In relish‐making, heat treatments and powdered spices used as ingredients may also have contributed to the reduction of β‐carotene. Likewise, cooking sweet potato leaves above 5 min and cilantro above 10 min decreased β‐carotene (Kao et al., 2014). The β‐carotene level reported in this study is lower than the decrease of β‐carotene content by 24.2% of cooked, fermented tomatoes (Bartkiene et al., 2015).

A recent study on human beings showed that the alcoholic fermentation of orange juice had increased the bioavailability of carotenoids (Hornero‐Méndez et al., 2018). The bioavailability of carotenoids is dependent on the carotenoid itself (polarity, conjugated double bonds), the food matrices (treatments undergone, presence or co‐ingestion of fat, structural characteristics with particular relevance of the type of predominant chromoplast, association of carotenoids with proteins), and the individual (including diseases, dietary patterns, lifestyle habits, gender, age, microbiota, and genetic factors) (Mapelli‐Brahm et al., 2020).

**FIGURE 2:**
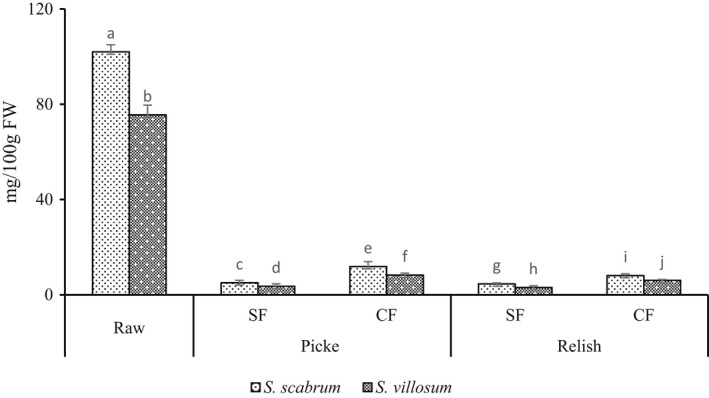
Vitamin C content of fermented African nightshade (ANS) pickle and relish products. Pickle (*S. scabrum* spontaneous fermented pickle (SsSFP), *S. villosum* spontaneous fermented pickle (SvSFP), *S. scabrum* controlled fermented pickle (SsCFP), and *S. villosum* controlled fermented pickle (SvCFP)) and relish (*S. scabrum* spontaneous fermented relish (SsSFR), *S. villosum* spontaneous fermented relish (SvSFR), *S. scabrum* controlled fermented relish (SsCFR), and *S*. *villosum* controlled fermented relish (SvCFR)). Whereas, Ss, *Solanum scabram*; Sv, *Solanum villosum*; CF, controlled fermentation; SF, spontaneous fermentation. Means ± *SD* (*n* = 3) with different superscript letters represent significant difference at *p* < .05

On the other hand, LAF significantly decreases vitamin C content in pickle and relish products (Figure 2). The results showed substantial vitamin C loss from fermented pickles (88.33%–95%) and relish products (91.93%–95.90%). Wafula (2017) reported vitamin C reduction by 81% and 66% in ANS, spontaneously and controlled fermentation. In this study, vitamin C losses in SF account for 91.66%–95% and 95.49%–95.90% in pickles and relish products, respectively. In addition, vitamin C losses in CF were similar to 88.33%–89.02% for pickles and 91.93%–92.06% for relish products, respectively. Vitamin C loss can be enhanced by extended storage, washing of vegetables, higher temperatures, low relative humidity, physical damage, and chilling injury (Lee & Kader, 2000; Wafula, 2017). Ascorbic acid (AA) is easily oxidized in aqueous solutions, favored by the presence of heavy metal ions (Cu^2+^, Ag^+^, and Fe^3+^), oxygen, alkaline pH, and high temperature (Lee & Kader, 2000). Oxidized AA, dehydroascorbic acid can be reduced to AA and irreversibly oxidized to form diketogulonic acid, with no vitamin C activity (Lee & Kader, 2000). Blanching prevents AA oxidase, phenolase, cytochrome oxidase, and peroxidase action, indirectly responsible for AA loss (Lee & Kader, 2000). However, blanching has been reported to decrease vitamin C content by 28%–82.4% due to dissolution and oxidation (Lee & Kader, 2000; Oboh, 2005). Furthermore, cooking has been reported to reduce vitamin C content by 30% and maintaining potatoes hot for 1 h further decreased vitamin C by 10% (Hägg et al., 1998; Lee & Kader, 2000). During fermentation, sucrose is converted to fructose and glucose, carbonyl groups of fructose react with vitamin C, reducing vitamin C content (Lee & Kader, 2000). Filannino et al. (2016) reported a reduction of vitamin C content after fermentation with *L. plantarum* and *L*. *brevis*. The remaining amount of vitamin C after fermentation is less to meet the recommended daily allowance of 100–200 mg (Lee & Kader, 2000). Likewise, vitamin C content in pickle and relish products was very low compared to the recommended dietary intake. Therefore, dietary diversification or fortification of the relishes is highly recommended.

Fermentation significantly increased the mineral content of the *S. scabrum* and *S. villosum* pickles and relish products compared to fresh leaves (Table 2). Sivakumar et al. (2020) reported Ca (442 mg/100 g DW) and Fe (12 mg/100 g DW) in fresh *S. villosum* to be lower than the values observed in this study. LAF improved the mineral content in pickles and relishes. Iron, Zn, Ca, S, P, Cu, and Ni content was improved by 0.58–2.01‐fold, respectively. Fermentation of amaranth leaves, pumpkin leaves, and capwood leaves showed increases in Ca, Mg, Zn, Fe, Se, and Cu (Ifesan et al., 2014). LAB improves nutritional quality and micronutrients’ bioavailability in food through antinutrients’ degradation by microbial enzymes. Therefore, LAF can be considered a strategy to enhance nutritional and functional quality by activating endogenous enzymes (Nkhata et al., 2018; Rollán et al., 2019) due to low pH. Also, LAB can produce different enzymes responsible for the hydrolysis of the food matrix into desirable nutritional and sensory quality. Despite sparsely available information, this study provides a finding for the mineral content of fermented *S. villosum* and *S. scabrum* pickles and relishes.

**TABLE 2:** Mineral content of fermented African nightshade (ANS) pickle and relish products

Relish products	P	S	Ca	Fe	Zn	Cu	Ni
**SsR**	1166.2 ± 15.0^a^	444.3 ± 4.2^a^	3392.6 ± 41.3^a^	185.5 ± 3.3.0^a^	8.11 ± 0.23^a^	2.24 ± 0.04^a^	0.29 ± 0.01^a^
SsSFP	1339.5 ± 14.1^b^	527.6 ± 9.7^b^	4259.3 ± 271.7^b^	230.6 ± 2.7^b^	15.4 ± 0.8^b^	4.50 ± 0.16^b^	0.38 ± 0.01^b^
SsSFR	1372.9 ± 3.6^c^	530.9 ± 26^b^	4692.6 ± 62.7^c^	268.0 ± 5.6^c^	21.4 ± 2.2^c^	5.3 ± 0.15^c^	0.49 ± 0.01^c^
SsCFP	1222.9 ± 12.6^d^	474.3 ± 14^c^	3792.6 ± 41.3^d^	198.0 ± 5.4^d^	12.4 ± 0.9^d^	3.47 ± 0.09^d^	0.32 ± 0.02^d^
SsCFR	1303.7 ± 6.8^e^	496.9 ± 8.5^d^	3984.0 ± 101.7^e^	217.6 ± 2.0^e^	17.4 ± 1.3^e^	3.94 ± 0.07^e^	0.37 ± 0.01^be^
**SvR**	1086.5 ± 9.5^f^	596.6 ± 4.97^e^	3113.7 ± 30.5^f^	142.7 ± 1.5^f^	5.53 ± 0.11^f^	1.95 ± 0.11^f^	0.26 ± 0.01^f^
SvSFP	1173.9 ± 5.6^a^	693.3 ± 1.8^f^	3447.1 ± 34.3^ag^	176.0 ± 6.2^g^	8.2 ± 0.7^a^	2.69 ± 0.3^g^	0.28 ± 0.24^a^
SvSFR	1205.5 ± 4.9^g^	736.6 ± 17.2^g^	3580.4 ± 35.6^g^	198.3 ± 6.5^d^	10.2 ± 0.7^g^	2.95 ± 0.06^h^	0.39 ± 0.01^be^
SvCFP	1102.6 ± 4.1^f^	660.0 ± 6.7^h^	3247.1 ± 34.2^af^	160.4 ± 1.7^h^	6.9 ± 0.7^af^	2.32 ± 0.15^a^	0.28 ± 0.01^af^
SvCFR	1131.6 ± 10^h^	696.6 ± 5.0^f^	3413.7 ± 87.8^ag^	179.3 ± 9.3^ag^	8.5 ± 0.9^ag^	2.79 ± 0.1^gh^	0.33 ± 0.01^d^

Note

Mean value (*n* = 3) ± *SD*. Means with different superscript letters within a column are significantly different *p* <.05. The results were expressed in mg/100g DW FW (dry weight) (fresh weight).

Abbreviations: SsR, *S. scabrum* raw; SsSFP, *S. scabrum* spontaneous fermented pickle; SsCFP, *S. scabrum* controlled fermented pickle; SvR, *S. villosum* raw; SvSFP, *S. villosum* spontaneous fermented pickle; SvCFP, *S. villosum* controlled fermented pickle.

### 3.3 Effect of fermentation on Chlorophyll and polyphenols

Fermentation significantly reduced Chlorophyll *a* content (*p* < .05) in SsSFP and Chlorophyll *b* in SsSFP, SsCFP, SvSFP, and SvCFP, respectively (Table 3). Also, fermentation reduced total Chlorophyll in all pickles (Table 3). During fermentation, acetic and lactic acid (LA) production degraded Chlorophyll *a* and *b* (Degrain et al., 2020). During fermentation, pH changes can produce new compounds such as Mg‐free Chlorophyll derivatives, a carotenoid with 5,8‐epoxide groups, brown pigments (*0*‐quinones), or phenolic compounds’ chemical oxidation masking the original green color of the leaves and reducing the Chlorophyll content (Ramírez et al., 2015).

**TABLE 3:** Chlorophyll content of fermented African nightshade (ANS) pickle and relish products

Pickle (g Kg^−1^) FW	Chlorophyll *a*	Chlorophyll *b*	Total Chlorophyll
SsR	29.80 ± 0.2^a^	27.95 ± 1.6^a^	57.75 ± 1.9^a2^
SsSFP	27.96 ± 0.8^a^	14.68 ± 0.8^c^	42.64 ± 1.6^c^
SsCFP	28.68 ± 2.3^a^	19.57 ± 5.5^b^	48.25 ± 3.3^d^
SsR	28.99 ± 0.2^a^	23.44 ± 0.6^ab^	52.43 ± 0.6^b^
SsSFP	22.13 ± 1.2^b^	9.71 ± 1.6 cd	31.85 ± 2.7^f^
SsCFP	29.04 ± 1.1^a^	13.44 ± 1.8^c^	42.48 ± 2.1^ce^

Note

Means ± *SD* (*n* = 3) with different superscript letters within a column are significantly different *p* < .05.

SsR, *S. scabrum* raw; SsSFP, *S. scabrum* spontaneous fermented pickle; SsCFP, *S. scabrum* controlled fermented pickle; SvR, *S. villosum* raw; SvSFP, *S. villosum* spontaneous fermented pickle; SsCFP, *S. villosum* controlled fermented pickle.

**FIGURE 3:**
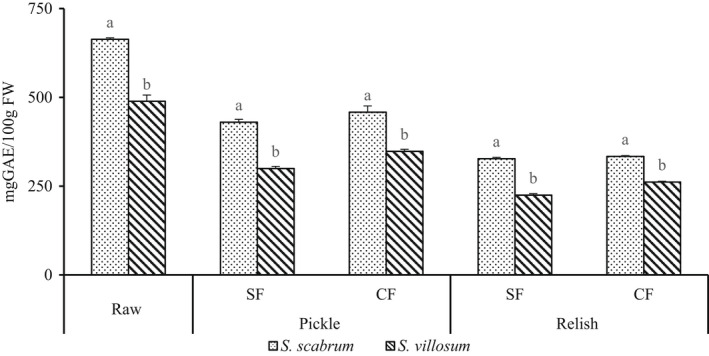
Total polyphenol content of fermented African nightshade (ANS) pickle and relish products. Pickle (*S. scabrum* spontaneous fermented pickle (SsSFP), *S. villosum* spontaneous fermented pickle (SvSFP), *S. scabrum* controlled fermented pickle (SsCFP), and *S. villosum* controlled fermented pickle (SvCFP)) and relish (*S. scabrum* spontaneous fermented relish (SsSFR), *S. villosum* spontaneous fermented relish (SvSFR), *S. scabrum* spontaneous fermented relish (SsCFR), and *S*. *villosum* controlled fermented relish (SvCFR)). Whereas, Ss, *Solanum scabram*; Sv, *Solanum villosum*; CF, controlled fermentation; SF, spontaneous fermentation. Means ± *SD* (*n* = 3) with different superscript letters represent significant difference at *p* < .05

On the other hand, LAF reduced the total phenolic content in Ss and Sv (Figure 3). Fermentation reduced polyphenol content by 61.29%–70.11% in pickles and 45.98%–53.51% in relish products, respectively (Figure 3). Both natural and commercial LAB reduced the total phenolic content, regardless of fermentation time. However, cooking pickles to relish further reduced polyphenol content in all relish products (Figure 3). In contrast, Degrain et al. (2020) reported an increase of total polyphenolic compounds in the fermented nightshade, from 6007.8 mg/kg (raw leaves) to 8016.8–8638.0 mg/kg fermented leaves with LAB strains of *Lactobacillus plantarum* (17a), *W. cibaria* (64), or *L. plantarum* (75). Furthermore, after fermentation, a reduction of total polyphenol content to 3822.5–5681.5 mg/kg *L. pseudomesenteroides* (56) and *Weissella cibaria* (21) was noticed. The decrease in total phenolic compounds in fermented nightshade leaves can be due to detoxification and utilization of phenolic acids as a carbon source (Filannino et al., 2016) by LAB. Subsequently, through phenolic acid decarboxylase, LAB can reduce total phenols by decarboxylation of ferulic and caffeic acids to other compounds such as 4‐vinyl phenol, 4‐vinyl guaiacol, or 4‐vinyl catechol. And further reduction to hydroxyphenyl propionic acids, such as dihydrocaffeic and dihydroferulic acids, could have affected (Degrain et al., 2020; Devi & Anu‐Appaiah, 2018). Similarly, *L. plantarum* decarboxylated most phenolic acids apart from gallic acid (Degrain et al., 2020). Additionally, *L. plantarum* metabolized caffeic, ferulic, p‐coumaric, and m‐coumaric acids out, which reduces the total polyphenol (Degrain et al., 2020).

### 3.4 Effect of fermentation on antinutritional factors

**FIGURE 4:**
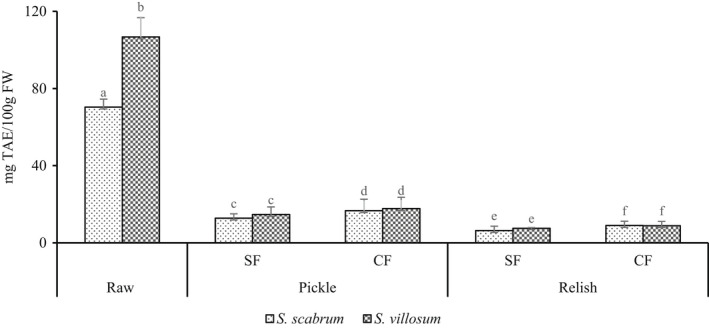
Tannin content in fermented African nightshade (ANS) pickle and relish products. Pickles (*S. scabrum* spontaneous fermented pickle (SsSFP), *S. villosum* spontaneous fermented pickle (SvSFP), *S. scabrum* controlled fermented pickle (SsCFP), and *S. villosum* controlled fermented pickle (SvCFP)) and relish (*S. scabrum* spontaneous fermented relish (SsSFR), *S. villosum* spontaneous fermented relish (SvSFR), *S. scabrum* controlled fermented relish (SsCFR), and *S. villosum* controlled fermented relish (SvCFR)). Whereas TAE, tannic acid equivalent; Ss, *Solanum scabram*; Sv, *Solanum villosum*; CF, controlled fermentation; SF, spontaneous fermentation. Means ± *SD* (*n* = 3) with different superscript letters represent significant difference at *p* < .05

**FIGURE 5:**
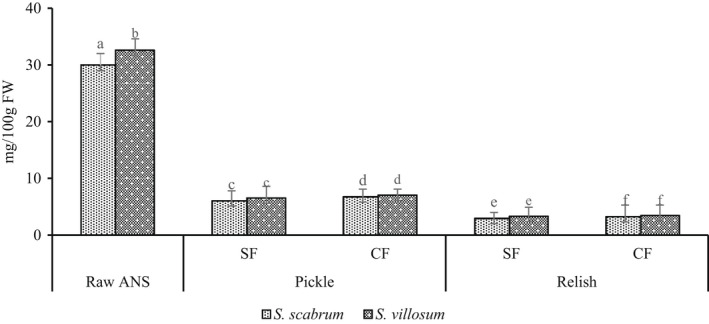
Oxalate content in fermented African nightshade (ANS) pickle and relish products. Pickle (*S. scabrum* spontaneous fermented pickle (SsSFP), *S. villosum* spontaneous fermented pickle (SvSFP), *S. scabrum* controlled fermented pickle (SsCFP), and *S. villosum* controlled fermented pickle (SvCFP)) and relish (*S. scabrum* spontaneous fermented relish (SsSFR), *S. villosum* spontaneous fermented relish (SvSFR), *S. scabrum* controlled fermented relish (SsCFR), and *S. villosum* controlled fermented relish (SvCFR)). Whereas; Ss, *S. scabrum*; Sv, *S. villosum*; CF, controlled fermentation; SF, spontaneous fermentation. Means ± *SD* (*n* = 3) with different superscripts represent significant difference *p* < .05

Lactic acid fermentation (LAF) substantially reduced tannins (76.27%–92.88%) and oxalate (77.33%–90%) levels of pickles and relish products, respectively (Figures 4 and 5). A 76.27%–86.23% reduction in tannins in pickles with a further decrease to 87.21%–92.88% in relishes was reported (Figure 4). Oxalate reduction also followed a similar trend, with a 77.33%–79.75% reduction in pickles and 89%–90% in relishes (Figure 5). LAB produces tannase and gallate decarboxylase enzymes used in the bioconversion of gallotanins and ellagitannins to increase phenol (gallic and ellagic acid), and reduce phenol (gallic and ell tannin) (Degrain et al., 2020). During fermentation, when pH decreases below 6 it contributes to the reduction of deprotonated divalent oxalate (C_2_O_4_^2−^) ion power to bind with divalent minerals such as Ca^2+^ ion to form insoluble oxalates (Simpson et al., 2009; Wadamori et al., 2014). Fermentation reduces oxalate, phytate, and tannins, which chelate minerals and hinder their bioavailability (Samtiya et al., 2021; Sangija et al., 2021). Despite several functional properties, tannins are toxic when chelating with minerals and cause a browning reaction, i.e., the darkening of foods (Sangija et al., 2021). Fermentation produces enzymes phytase and tannase that degrade the phytate and tannins and improve the availability of calcium, iron, and zinc (Samtiya et al., 2021). Similarly, eliminating toxins such as tannins, oxalates, and phytates through LAF ensures food safety. Therefore, reducing tannins and oxalates can ensure the safety and bioavailability of some mineral elements in pickles and relish products.

### 3.5 Consumer's acceptability of fermented relish

The majority of the consumers preferred SsCFR with a mean score of 7.96. Interestingly, all the three SvCFR, SsSFR, and SvSFR were equally preferred (Table 4). According to Wafula, 2017, 80% of the panelists showed preference toward fermented ANS. Odor and flavor were the attributes preferred mainly by the majority of the consumers, with a mean score of 7.81, while color was least preferred. Degrain et al. (2020) reported that LAF influenced the color of *Solanum retroflexum* leaves through the degradation of Chlorophyll *a* and *b*. Furthermore, low pH is associated with color destruction in fermented vegetables due to the production of acids, concurrently with findings (Table 3). In a study by Wafula (2017), a similar color mean score (7.9) was reported for fermented ANS. In addition, other parameters, such as texture, appearance, taste, saltiness, sourness, bitterness, and spiciness, were also ranked higher by consumers (Table 4). This suggests that, other than LAF, relish‐making further improved the sensory quality of the pickles. Likewise, LAF improved sensory attributes of color, taste, smell, appearance, and general acceptability (Wafula, 2017).

**TABLE 4:** Consumer's acceptability test scores of fermented African nightshade (ANS) relish products

Formulation		Color	Texture	Appearance	Taste	Odor	Flavor	Saltiness	Sourness	Bitterness	Spiciness	Overall acceptability
SsCFR	Min	1	2	2	2	2	2	2	2	2	2	2
	Max	9	9	9	9	9	9	9	9	9	9	9
	Mean	7.84^a^	7.86^a^	7.88^a^	7.90^a^	7.90^a^	7.90^a^	7.90^a^	7.94^a^	7.81^a^	7.94^a^	7.96^a^
	*SD*	0.996	0.84	0.84	0.83	0.796	0.78	0.91	0.87	0.97	0.81	0.783
SvCFR	Min	1	1	1	1	1	1	1	1	1	2	1
	Max	9	9	9	9	9	9	9	9	9	9	9
	Mean	7.59^b^	7.69^b^	7.71^b^	7.73^b^	7.76^b^	7.76^b^	7.67^b^	7.66^b^	7.60 ^a^	7.76^b^	7.79^b^
	*SD*	1.37	1.37	1.04	1.05	0.977	1.00	1.10	1.23	1.32	1.00	0.981
SsSFR	Min	2	2	2	2	2	2	2	2	2	2	2
	Max	9	9	9	9	9	9	9	9	9	9	9
	Mean	7.75^ab^	7.79^ab^	7.80^ab^	7.80^ab^	7.80^ab^	7.81^ab^	7.73^bc^	7.71^bc^	7.77 ^a^	7.81^ab^	7.82^b^
	*SD*	1.03	0.93	0.88	0.94	0.914	0.91	1.07	1.1	0.86	0.91	0.931
SvSFR	Min	1	2	2	2	2	2	1	1	1	2	2
	Max	9	9	9	9	9	9	9	9	9	9	9
	Mean	7.71^ab^	7.77^ab^	7.78^ab^	7.76^cb^	7.78^ab^	7.78^ab^	7.73^bd^	7.67^bd^	7.73 ^a^	7.78^b^	7.81^b^
		1.22	0.95	0.97	1.05	0.94	0.96	1.07	1.25	1.06	0.96	0.95
Overall acceptability of each parameter	Mean	7.72	7.78	7.79	7.80	7.81	7.81	7.76	7.75	7.73	7.82	
	*SD*	1.15	1.02	0.93	0.97	0.91	0.91	1.04	1.11	1.05	0.92	

Note

Means with different superscript letters within a column are significantly different *p* < .05, *n* = 370).

1 = dislike extremely, 5 = neither like nor dislike, 9 = like extremely; SsCFR, *S. scabrum* controlled fermented relish; SvCFR, *S. villosum* controlled fermented relish; SsSFR, *S. scabrum* spontaneous fermented relish; SvSFR, *S. villosum* spontaneous fermented relish.

### 3.6 Shelf life of relish products during storage

Results of the shelflife study of the LAF fermented relish products are presented in Table 5 and Table 6. After six months of room and refrigeration storage of the fermented relish products, no change in pH and TTA was recorded. Also, no coliforms, total bacteria, *Lactobacillus*, yeast, and mold were detected (Tables 5 and 6). Likewise, Wafula (2017) reported that due to the absence of fermented ANS in a starter culture, some microbes were detected in the spontaneously fermented product after storage (10°C and 25°C). Microbiological results indicated that the relish product is safe for consumption. Low pH (3.2–3.3) inhibited the growth of pathogens (Wafula, 2017). Heat treatment of the pickle to make relishes killed microorganisms in the fermented pickles, including *Lactobacillus*, which hinders their growth in the relish product (Sangija et al., 2021). Fermentation of *S. scabrum* with *L. plantarum* and *Leuconostoc mesenteroides* inhibited *Listeria monocytogenes* and *Salmonella enterica* serovar. Enteritidis (Sivakumar et al., 2020). Therefore, from the microbiological quality point of view, all relishes were shown to be safe after six months of storage at 4°C and 27°C.

**TABLE 5:** Shelf life of fermented African nightshade (ANS) relish products stored at ambient storage (27°C)

Time (months)	0				1				2				3				4				5				6			
Relishes	Ss		Sv		Ss		Sv		Ss		Sv		Ss		Sv		Ss		Sv		Ss		Sv		Ss		Sv	
Fermentation	SF	CF	SF	CF	SF	CF	SF	CF	SF	CF	SF	CF	SF	CF	SF	CF	SF	CF	SF	CF	SF	CF	SF	CF	SF	CF	SF	CF
pH	3.3	3.2	3.3	3.2	3.3	3.2	3.3	3.2	3.3	3.2	3.3	3.2	3.3	3.2	3.3	3.2	3.3	3.2	3.3	3.2	3.3	3.2	3.3	3.2	3.3	3.2	3.3	3.2
TTA	0.7	0.6	0.6	0.6	0.7	0.6	0.6	0.6	0.7	0.6	0.6	0.6	0.7	0.6	0.6	0.6	0.7	0.6	0.6	0.6	0.7	0.6	0.6	0.6	0.7	0.6	0.6	0.6
Total bacteria	‐	‐	‐	‐	‐	‐	‐	‐	‐	‐	‐	‐	‐	‐	‐	‐	‐	‐	‐	‐	‐	‐	‐	‐	‐	‐	‐	‐
Yeast & mold	‐	‐	‐	‐	‐	‐	‐	‐	‐	‐	‐	‐	‐	‐	‐	‐	‐	‐	‐	‐	‐	‐	‐	‐	‐	‐	‐	‐
Coliform	‐	‐	‐	‐	‐	‐	‐	‐	‐	‐	‐	‐	‐	‐	‐	‐	‐	‐	‐	‐	‐	‐	‐	‐	‐	‐	‐	‐
LAB	‐	‐	‐	‐	‐	‐	‐	‐	‐	‐	‐	‐	‐	‐	‐	‐	‐	‐	‐	‐	‐	‐	‐	‐	‐	‐	‐	‐
Vitamin C	4.6	8.1	3.1	6.1	3	6	2.1	4.2	2	4.2	1.8	3.9	1.5	3.6	1.4	3.5	1.2	3.1	1	3.1	0.7	2.5	0.6	2.1	0.4	1.6	0.4	1.8
% loss vit.C	0	0	0	0	35	26	32	31	57	48	42	36	67	56	55	43	74	62	68	49	85	69	81	66	91	80	87	70
β‐carotene	121	79	148	97	110	71	131	89	101	62	122	78	89	55	115	69	69	41	100	54	60	35	87	41	54	30	80	38
% loss of β‐carotene	0	0	0	0	9	10	11	8	17	22	18	20	26	30	22	29	43	48	32	44	50	56	41	58	55	62	46	61

Note

Whereas: SS, *S. scabrum*; Sv, *S. villosum*; SF, spontaneous fermentation; CF, controlled fermentation; Vit. C, Vitamin C; ‐, nil. Vitamin C and β‐carotene were presented in mg/100 gFW (fresh weight).

Abbreviations: TTA, titratable acidity; LAB, lactic acid bacteria.

**TABLE 6:** Shelf life of fermented African nightshade (ANS) relish products stored under refrigeration temperature (4°C)

Months	0				1				2				3				4				5				6			
Relishes	Ss		Sv		Ss		Sv		Ss		Sv		Ss		Sv		Ss		Sv		Ss		Sv		Ss		Sv	
Fermentation	SF	CF	SF	CF	SF	CF	SF	CF	SF	CF	SF	CF	SF	CF	SF	CF	SF	CF	SF	CF	SF	CF	SF	CF	SF	CF	SF	CF
pH	3.3	3.2	3.3	3.2	3.3	3.2	3.3	3.2	3.3	3.2	3.3	3.2	3.3	3.2	3.3	3.2	3.3	3.2	3.3	3.2	3.3	3.2	3.3	3.2	3.3	3.2	3.3	3.2
TTA	0.7	0.6	0.6	0.6	0.7	0.6	0.6	0.6	0.7	0.6	0.6	0.6	0.7	0.6	0.6	0.6	0.7	0.6	0.6	0.6	0.7	0.6	0.6	0.6	0.7	0.6	0.6	0.6
Total bacteria	‐	‐	‐	‐	‐	‐	‐	‐	‐	‐	‐	‐	‐	‐	‐	‐	‐	‐	‐	‐	‐	‐	‐	‐	‐	‐	‐	‐
Yeast & mold	‐	‐	‐	‐	‐	‐	‐	‐	‐	‐	‐	‐	‐	‐	‐	‐	‐	‐	‐	‐	‐	‐	‐	‐	‐	‐	‐	‐
Coliform	‐	‐	‐	‐	‐	‐	‐	‐	‐	‐	‐	‐	‐	‐	‐	‐	‐	‐	‐	‐	‐	‐	‐	‐	‐	‐	‐	‐
LAB	‐	‐	‐	‐	‐	‐	‐	‐	‐	‐	‐	‐	‐	‐	‐	‐	‐	‐	‐	‐	‐	‐	‐	‐	‐	‐	‐	‐
Vitamin C	4.6	8.1	3.1	6.1	4.3	7.8	3.0	5.8	4.0	7.5	2.8	5.5	3.7	7.2	2.3	5.2	3.5	7.0	2.1	4.9	3.2	6.7	1.8	4.5	3.0	6.3	1.3	4.1
% loss vit. C	0	0	0	0	7	4	3	5	13	7	10	10	20	11	26	15	24	14	32	20	30	17	42	26	35	22	58	33
β‐carotene	121	79	148	97	118	74	140	92	113	70	136	88	110	68	128	84	106	63	121	80	101	60	116	76	97	56	112	71
% loss of β‐carotene	0	0	0	0	2	6	5	5	7	11	8	9	9	14	14	13	12	20	18	18	17	24	22	22	20	29	24	27

Note

Whereas: Ss, *S. scabrum*; Sv, *S. villosum*; SF, spontaneous fermentation; CF, controlled fermentation; Vit. C, Vitamin C; ‐, nil. Vitamin C and β‐carotene were presented in mg/100 g.

Abbreviations: TTA, titratable acidity; LAB, lactic acid bacteria.

Results on a gradual loss in vitamin C in relishes stored at ambient (27°C) and refrigeration (4°C) temperatures are presented in Tables 5 and 6. Vitamin C losses were high at ambient temperature than the refrigeration temperature. Ambient storage at 27°C and the transparent PET packaging allow light intensity, facilitating the denaturation of vitamin C (Lee & Kader, 2000). A low temperature of 4°C with no light intensity effect supports the low losses in vitamin C in refrigeration storage.

On the other hand, β‐carotene losses amount to 46%–62% and 20%–29% for ambient and refrigeration storage for the relishes (Tables 5 and 6). Despite the reduction of β‐carotene, still the remaining quantity is sufficient to contribute to the recommended daily allowance (adult 75–180 mg and children 30–150 mg) (IBM Watson Health, 2022). This agrees with Singh et al. (2013) that, a high loss of β‐carotene in room storage was 85% for glass bottles and 81% for plastic bottles. Oxidation is the leading cause of carotenoid degradation in foods. However, in processed foods, the oxidation mechanism is complex but is facilitated by moisture, temperature, presence of prooxidants, antioxidants, and lipids (Singh et al., 2013).

## 4 CONCLUSION

Lactic acid fermentation (LAF) enhanced ANS pickle and relish products’ nutritional, sensory, and safety quality. Specifically, LAF improved β‐carotene and minerals contents but reduced vitamin C, total phenols, and Chlorophyll levels. LAF also reduced tannins and oxalate. The *SsCFRP* product was the most preferred over other relish products. All relish products were stable at ambient and refrigeration temperatures after six months of storage, hence contributing to product safety. Therefore, LAF can be recommended as the best preservation method for African nightshades (ANSs). From the study, LAF has been shown to retain nutrients, improve safety and shelf life, offer diverse products, subsequently contributing to minimize postharvest losses.

## CONFLICTS OF INTEREST

The authors declare no conflict of interest.

## AUTHOR CONTRIBUTION

**Frank Sangija:** Conceptualization (lead); Data curation (lead); Formal analysis (lead); Methodology (lead); Resources (supporting); Software (lead); Validation (equal); Writing – original draft (lead); Writing – review & editing (equal). **Haikael Martin:** Conceptualization (supporting); Data curation (supporting); Formal analysis (supporting); Funding acquisition (supporting); Investigation (supporting); Methodology (supporting); Project administration (supporting); Resources (supporting); Software (supporting); Supervision (supporting); Validation (supporting); Writing – original draft (supporting); Writing – review & editing (supporting). **Athanasia Matemu:** Conceptualization (equal); Data curation (supporting); Formal analysis (supporting); Funding acquisition (supporting); Investigation (lead); Methodology (supporting); Project administration (supporting); Resources (supporting); Software (supporting); Supervision (lead); Validation (equal); Writing – original draft (supporting); Writing – review & editing (lead).
